# Gene Encoding Chitinase 3-Like 1 Protein (CHI3L1) is a Putative Oncogene

**Published:** 2011-09

**Authors:** Vadym M. Kavsan, Vladimir P. Baklaushev, Olena V. Balynska, Anton V. Iershov, Pavlo O. Areshkov, Gaukhar M. Yusubalieva, Nadezhda Ph. Grinenko, Ilya V. Victorov, Vadym I. Rymar, Marc Sanson, Vladimir P. Chekhonin

**Affiliations:** 1 *Department of Biosynthesis of Nucleic Acids, Institute of Molecular Biology and Genetics, National Academy of Sciences of Ukraine, 150 Zabolotnogo Street, Kyiv, Ukraine;*; 2 *Laboratory of Immunochemistry, V.P. Serbsky National Research Centre for Social and Forensic Psychiatry, RUSA Ministry of Health, Moscow, Russia;*; 3 *Chair of Medical Nanobiotechnology N.I. Pirogov Russian State Medical University, Moscow, Russia;*; 4 *AP-HP, Groupe Hospitalier Pitié-Salpêtrière, Service de Neurologie Mazarin and UMR 975 INSERM-UPMC, Paris, France*

**Keywords:** glioblastoma, chitinase 3-like 1 protein (CHI3L1), oncogene

## Abstract

An important task in understanding oncogenesis is the identification of those genes whose copy number and expression increase during tumorigenesis. Previously, in an effort to identify genes which could be used as molecular markers for glial tumors, we compared gene expression in glioblastoma to the normal brain cells. Among the genes with the most pronounced increased expression in tumors there was *CHI3L1*, encoding the secreted chitinase 3-like 1 protein (also known as HC gp-39 or YKL-40). Expression of CHI3L1 was found increased significantly in various tumors in comparison with corresponding normal tissues. Here we show that *CHI3L1* can decrease the doubling time of 293 cells. We have also demonstrated that CHI3L1 allows the anchorage-independent growth in soft agar and, in addition, stable *CHI3L1* expression made 293 cells tumorigenic: these cells stimulate the initiation of tumors after their xenograft transplantation into the Wistar rat brains. Thus, the overexpression of *CHI3L1* is likely to be critical in the development of some tumors and when we gain more information about mechanisms of CHI3L1 oncogenicity, it could be used as one of the potential targets for anticancer therapy.

## INTRODUCTION

Previously, in an effort to identify genes which could be used as molecular markers for glial tumors, we compared gene expression in glioblastoma, the most aggressive human brain tumor, to the normal brain cells and revealed 44 genes with more than 5-fold higher expression level in tumors. One of such genes overexpressed in glioblastoma was *CHI3L1*, encoding the secreted chitinase 3-like 1 protein (1-3). The expression of *CHI3L1* gene was found in synovial cells, articular cartilage chondrocytes and increased significantly in various tumors, and cell lines derived from such tumors, including tumors of the bone, brain, breast, lung, and ovary (4).

CHI3L1 is a member of the 18 glycosyl hydrolase family mapped at 1q32 genomic region, which is frequently amplified in different human cancers; amplifications were documented for glioma, esophageal squamous cell carcinoma, retinoblastoma (5-7). 1q32 locus contains about 160 protein-coding genes (http://www.ncbi.nlm.nih.gov/gene). Among them, mouse double minute-4 p53 binding homolog (*MDM4*) and contactin 2 (*CNTN2*) are amplified in a subset of malignant gliomas without TP53 mutation or *MDM2* amplification (5), *SMYD2* is amplified in esophageal squamous cell carcinoma (6), and *KIF14* is amplified in breast cancer (7). However, copy number profiling for human glioma does not show *CHI3L1* gene amplification (8). It was reported that polymorphism in the promoter region of *CHI3L1* disrupts MYC/MAX binding site (9), which may lead to decreased expression. However, our recent studies revealed no correlation between this polymorphism and gene expression in glioblastoma (10). Thus, it is possible that *CHI3L1* overexpression is explained by some other mechanisms, for example, by presence of distant regulators affected by amplification in this or other types of tumors.

It was demonstrated that CHI3L1 has growth promoting activity and can initiate mitogen-activated kinase (MAPK) and phosphoinositide 3-kinase (PI-3K) signaling cascades in human connective tissue cells, leading to the phosphorylation of extracellular signal-regulated kinase 1 and 2 (ERK1/2) and AKT, respectively (11). In another study, the stimulation of human articular chondrocytes or skin fibroblasts with interleukin-1 or tumor necrosis factor alpha (TNFα) in the presence of CHI3L1 protein reduced the phosphorylation of both p38 and JNK1/2 (12). *CHI3L1* expression had a positive association with the expression of phosphorylation of ERK1/2, which was strongly correlated with a poor response to radiotherapy and a negative clinical outcome (13). It was detected an obvious decrease in phospho-ERK1/2 in *CHI3L1* siRNA-transfected U87 cells accompanied by reduced cell proliferation after *CHI3L1* silencing (14).

In the current study we demonstrate the oncogenic properties of *CHI3L1* gene: its stable expression allows anchorage-independent cell growth in soft agar, decreases the doubling time of 293 cells and makes 293 cells tumorigenic being xenografted into the Wistar rat brains.

## METHODS

### Cells

293 cells (Human Embryonic Kidney 293 cells, also often referred to HEK 293, or less precisely as HEK cells) were kindly provided by Prof. V. Filonenko; 293 cells, stably transfected with pcDNA3.1 and pcDNA3.1/GFP were kindly provided by Dr. V. Grishko (Department of Cell Signaling, Institute of Molecular Biology and Genetics, Kyiv, Ukraine). Cells were grown in Dulbecco’s modified Eagle’s medium (DMEM) (Sigma, San Diego, CA, USA) supplemented with 10% fetal bovine serum (FBS) (Sigma) and 100 μg/ml penicillin/100 u/ml streptomycin (Sigma) in an environment of 95% air/5% CO_2_.

### Gene expression in glioma

Gene expression data for 225 glioblastoma and 74 normal brain samples were obtained from the Gene Expression Omnibus (GEO) repository (http://www.ncbi.nlm.nih.gov/geo). To compare data from different experiments we used normalization method proposed for real-time polymerase chain reaction (PCR) (15) and normalized all data by dividing expression level of every gene on geometric average of three housekeeping genes: β-actin (*ACTB*), glyceraldehyde-3-phosphate dehydrogenase (*GAPDH*), and TATA-box binding protein (*TBP*). Comparison between two groups was done by using the independent samples t test. A value of *P*<0.05 was considered as statistically significant.

### Proteins and antibodies

Native CHI3L1 was purified from supernatant of conditioned MG-63 cell medium as described by Harvey *et al*. (16).

Goat polyclonal antibodies against human CHI3L1 (S-18) were purchased from Santa Cruz Biotechnology (Santa Cruz, CA, USA), horse-radish peroxidase anti-goat immunoglobulin conjugates used in Western blot, goat anti-rabbit Alexa Fluor 488 and rabbit anti-goat Alexa Fluor 488 antibodies used in immunofluorescence were from Invitrogen (Carlsbad, CA, USA).

### Immunofluorescence and confocal microscopy

293 cells were seeded on coverslips and allowed to grow to near-confluence. The cells were washed in cold phosphate-buffered saline (PBS), fixed with 3.7% paraformaldehyde, washed three times for 5 min each with PBS and blocked with 5% horse serum (Sigma) in PBS (blocking buffer) for 30 min at room temperature. Incubations were performed at room temperature with antibodies diluted in blocking buffer. Slides were mounted using PVA-DABCO (Sigma) and images were captured with a Zeiss LSM 510 Meta confocal microscope (Hamburg, Germany).

### Cell proliferation assay of CHI3L1-treated 293 cells

Cell count was performed to assay the cell growth curve in CHI3L1-treated 293 cells. Briefly, an equal number of cells (5 × 10^4^) were seeded in 6-well cultural plates in DMEM containing 10% FBS, and then starved for 1 day, before being treated with CHI3L1 (100 ng/ml). The cells were then washed and trypsinized, and the number of cells was assessed using a hemocytometer (Neubauer Improved, Marienfeld, Germany). The data are expressed as the mean ± SEM for three independent experiments.

### Generation of 293 cell line, stably expressing CHI3L1

The 1.0-kb Hind III/Bam HI fragment of CHI3L1 cDNA was ligated to the Bam HI/Hind III site of the pcDNA3.1(+)/neomycin mammalian expression vector (Invitrogen). To check the transfection effectiveness, after 1 day of seeding, 293 cells were transfected by 5 μg of pcDNA3.1_*CHI3L1* using 8 μl jetPEI (Polyplus, New York, NY, USA) per 60-mm dish. CHI3L1 levels were visualized by Western blot and immunofluorescense.

To obtain a cell line stably producing CHI3L1 protein, 293 cells were transfected by 5 μg pcDNA3.1_CHI3L1 and 8 μl jetPEI per 60-mm dish. 0.8 mg/ml geneticin G418 sulphate (Sigma) was used to select transfectants after 3 weeks.

### Cell proliferation assay of 293 cells, stably expressing *CHI3L1*

293 cells stably expressing *CHI3L1*, and 293 cells, stably transfected with pcDNA3.1 were seeded in quadruplicates in 96 well plate at density 1 × 10^3^ cells/well and grown in DMEM, supplemented with 10% FBS, geneticin G418 sulphate and 100 μg/ml penicillin/100 u/ml streptomycin for 7 days. Cell proliferation was measured using MTT (Sigma) at days 1, 2, 3, 5, 6 and 7 of the seeding.

### Soft agar colony formation assay

For soft agar assay, we placed 1 × 10^4^ 293 cells, stably expressing *CHI3L1*, 293 cells, stably transfected with pcDNA3.1 or 293 cells, stably expressing *GFP* in 2 ml of 0.35% low gelling temperature agarose (Gibco, New York, NY, USA) in DMEM supplemented with 10% FBS and overlaid them on 2 ml of 0.8% agarose/10%FBS/DMEM in a 35-mm diameter dish. We grew cells at 37°C for 21 days to allow colony formation. Colonies were visualized by staining with 0.005 % crystal violet. The experiment was performed three times.

### Xenograft models

All procedures were conducted in accordance with institutional regulations for use of laboratory animals. Adult female Wistar rats (200-220 g, n=10) were used as recipients for 293 cells, stably transfected with pcDNA3.1 or 293 cells stably expressing *CHI3L1*. 4 × 10^5^ cells were injected stereotactically under ketamine anesthesia (Narishige Stereotactic Apparatus, Japan) into the caudoputamen at the following stereotactic coordinates: Ap –1, L 3.0, V 4.5, and TBS –2.4 mm according to the Rat Brain Atlas. The cells were injected with a Hamilton microsyringe connected to an infusomat at the rate of 3 μl/min (5-10 μl). Animals were killed at 21 day postinjection. Tumor volume (*V*) was estimated from the length (*l*) and width (*w*) of the tumor using the formula: *V* = (π/6) × ((*l* + *w*)/2)^3^. Tumors were fixed in formalin overnight at 4°C and embedded in paraffin. Tissue sections were de-waxed and stained with hematoxylin and eosin.

## RESULTS

**Figure 1:**
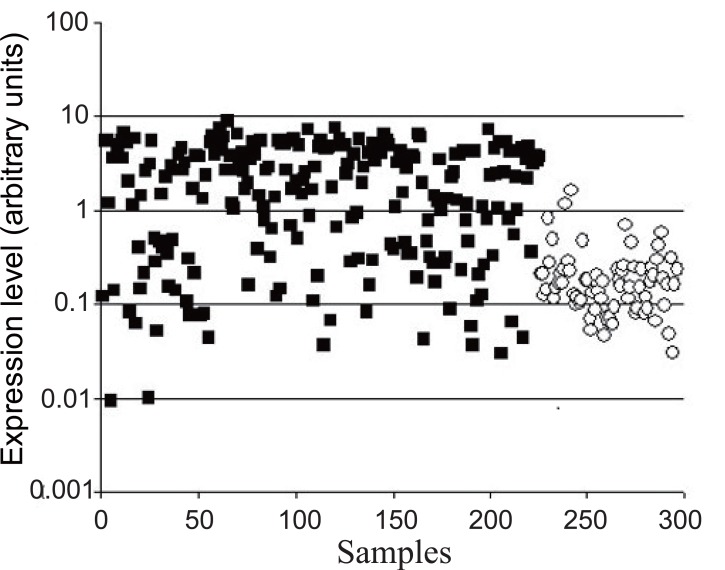
*CHI3L1* gene expression in glioblastomas and normal brain. Data according to GEO repository (http://ncbi.nlm.nih.gov/GEO), log coordinates. Comparison between two groups was done by using the independent samples t test, *P*<0.001. ■, Individual glioblastoma sample, ○, individual normal brain sample.

Previously, significantly increased *CHI3L1* gene expression was shown for a number of tumors (4) including glioblastomas in our and other laboratories (1-3). These results were obtained mostly by SAGE, Northern and Western blot analyses or PCR using comparably small number of samples. The data from Gene Expression Omnibus (GEO) repository (http://www.ncbi.nlm.nih.gov/geo) obtained by microarray approach allow comparing gene expression levels in 225 glioblastoma and 74 normal brain samples. The significant difference between *CHI3L1* gene expression levels in glioblastoma in comparison to normal brain samples, obtained by this approach, is shown in Fig. 1. In the same time, it is possible to notice the significant overlap in the levels in individual samples of tumor and normal brain. As it was shown recently, *CHI3L1* expression is absent in subset of glioblastomas termed “proneural”, which comprise up to 30% of all glioblastomas, due to promoter hypermethylation (17).

**Figure 2:**
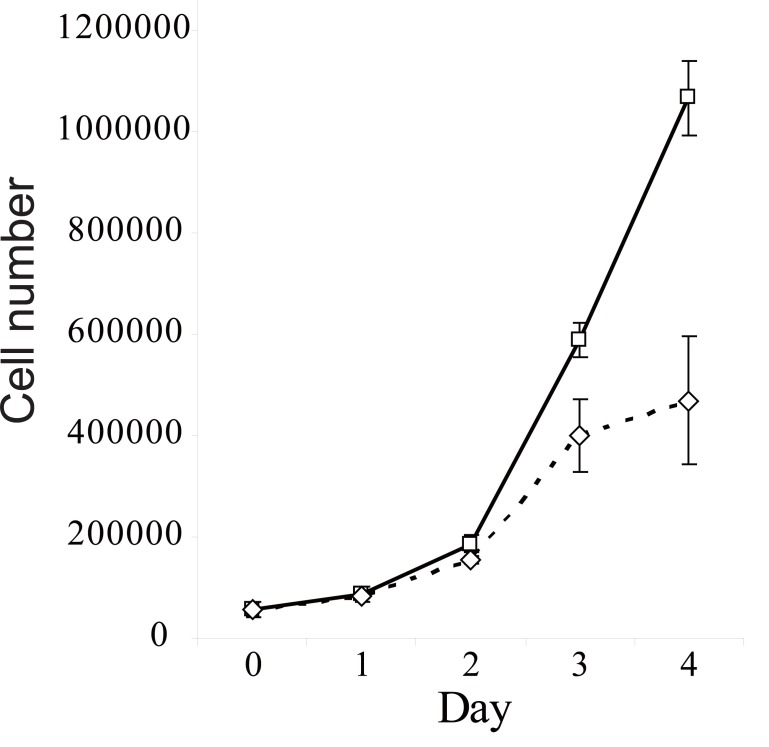
Effect of *CHI3L1* on proliferation of 293 cells. Starved 293 cells were incubated in unsupplemented medium (▬□▬) or in medium containing 100 ng/ml *CHI3L1* (־◊־) for time periods indicated, followed by cell harvesting and counting as described. Each individual point represents the mean of the values obtained from triplicate experiments.

Proliferative response of cells on treatment by CHI3L1 was studied with 293 cell line (Human Embryonic Kidney 293 cells, also often referred to as HEK 293 cells), as these cells do not produce CHI3L1 under any culture conditions. Obtained results are shown in Fig. 2, demonstrating increased cell number in culture maintained in the presence of CHI3L1 compared with cells maintained in unsupplemented medium.

Given that *CHI3L1* is overexpressed in many human cancer cells, we addressed the question of whether CHI3L1 possesses transforming properties on 293 cells, one of the most common cell types used in molecular biology research. To this end, we used the expression construct pcDNA3.1/*CHI3L1* under the control of cytomegalovirus (CMV) promoter to establish the 293-*CHI3L1* cell line from parental 293 cells. Overexpression of *CHI3L1* on the protein level in this cell line was confirmed by immunofluorescence and Western blot analysis.

**Figure 3:**
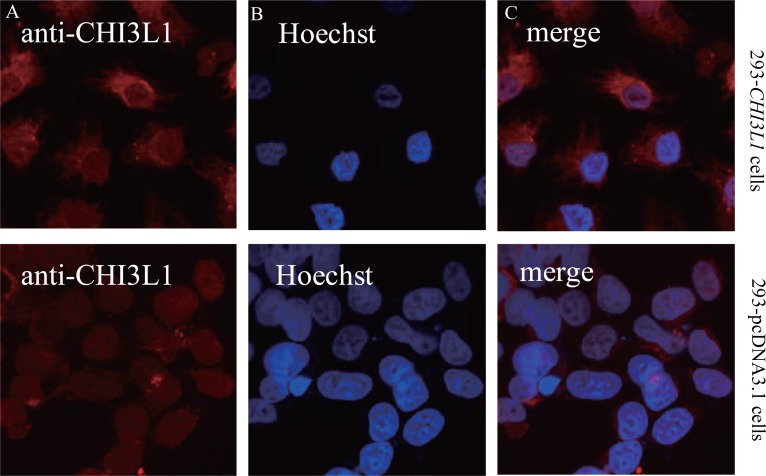
Immunofluorescent analysis of *CHI3L1*. (A) Paraformaldehyde fixed 293 cells and 293 cells stably expressing *CHI3L1* with polyclonal antibodies to *CHI3L1*; (B) cell nuclei – Hoechst 33342 staining; (C) the merged image indicates *CHI3L1* localization in cytoplasm of 293 cells stably expressing *CHI3L1*. Red fluorescence: rabbit anti-goat Alexa Fluor 594.

Immunofluorescent analysis of CHI3L1 in paraformaldehyde fixed cells with polyclonal antibodies to CHI3L1 indicated that the protein was localized diffusely in cytoplasm of 293 cells stably expressing *CHI3L1* (Fig. 3), corresponding to the localization of CHI3L1 protein in breast cancer cells (18).

**Figure 4:**
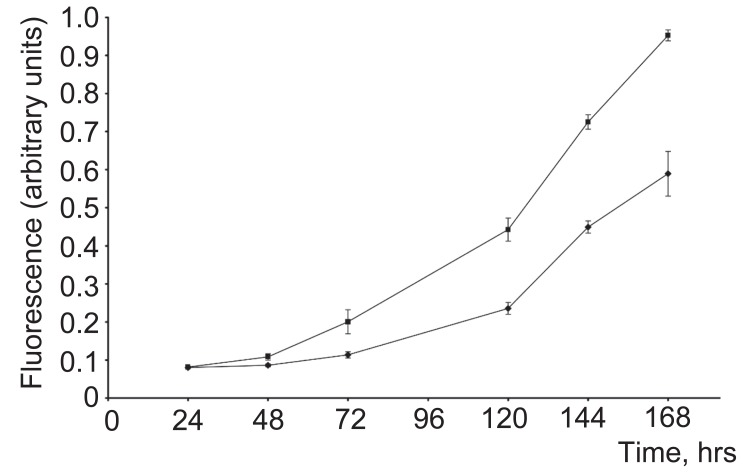
Proliferation of 293 cells stably expressing *CHI3L1*. –♦– 293 cells, stably expressing *CHI3L1*, –▲– 293 cells, transfected with empty vector. Proliferation was measured by fluorescence after 3.5 hrs of exposure of the cells to MTT reagent. The data shown are the means±S.D. from four experiments for each cell line.

*CHI3L1*-expressing 293 cells had an accelerated growth rate relatively to the parental 293 controls. Four days after equal numbers of cells were plated, there were approximately 1.5 times as many *CHI3L1*-expressing cells as parental cells (Fig. 4).

**Figure 5:**
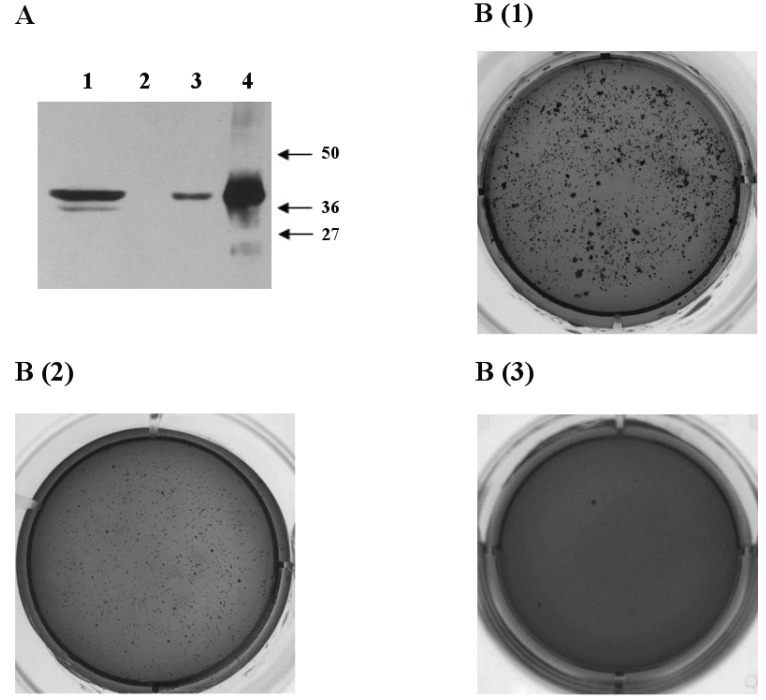
Oncogenic properties of *CHI3L1*. (A) Western blotting: total lysates of 293 cells stably expressing CHI3L1 (1), total lysates of 293 cells (2), recombinant CHI3L1 (3), glioblastoma total lysate, prepared earlier [19] (4); (M) prestained protein MW marker SM0441; (B) 293 cells in a soft agar colony formation assay (1•10^4^ cells\per well) stably transfected by pcDNA3.1\*CHI3L1* (1), pcDNA3.1/*GFP* (2) and pcDNA3.1 (3).

Next, we used a soft agar transformation assay that measures whether cells can undergo anchorage-independent growth, which is one of the most consistent indicators of oncogenic transformation. After 3 weeks, 293-*CHI3L1* cells grew into robust colonies in soft agar, a property not observed in the cells transfected with empty vector or in the cells transfected with vector that stably produced the green fluorescent protein (GFP). As it is shown in Fig. 5, 293-*CHI3L1* cells have much higher capacity to generate colonies than control cells after 10-12 days in the colony assay formation conditions.

**Figure 6:**
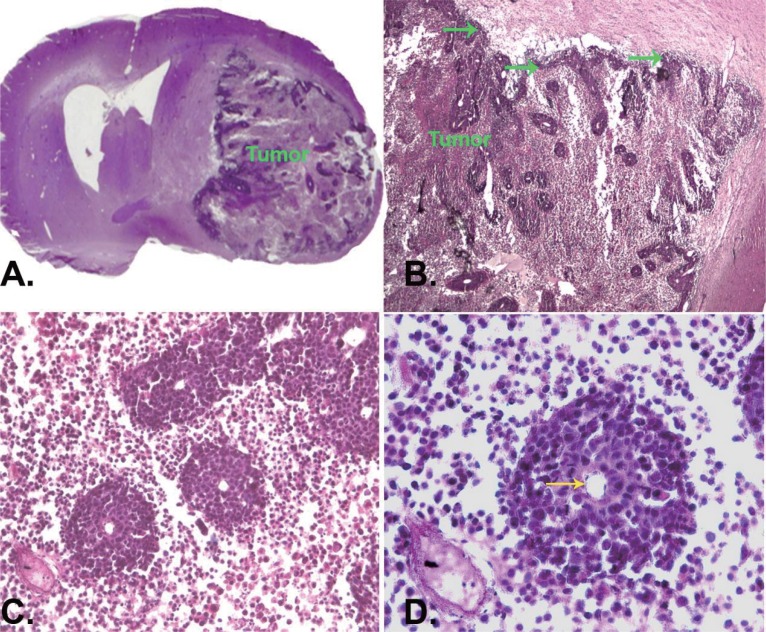
Histological analysis of intracerebral implanted 293-*CHI3L1* cells. (A) Rat brain tumor formed by intracranial inoculation of 293 cells stably expressing *CHI3L1*. Displacement of median cerebral structures and hydrocephaly in contralateral hemisphere. Tumors were characterized by lobular structure with dense superficial cell layer (B, green arrows) and prominent lobules (100-150 μm) and central newly ingrowing blood vessels (C, D, yellow arrow). Haematoxylin staining of paraffin embedded slices. Magnification ×5 (A), ×20 (B), ×100 (C), ×400 (D).

**Figure 7:**
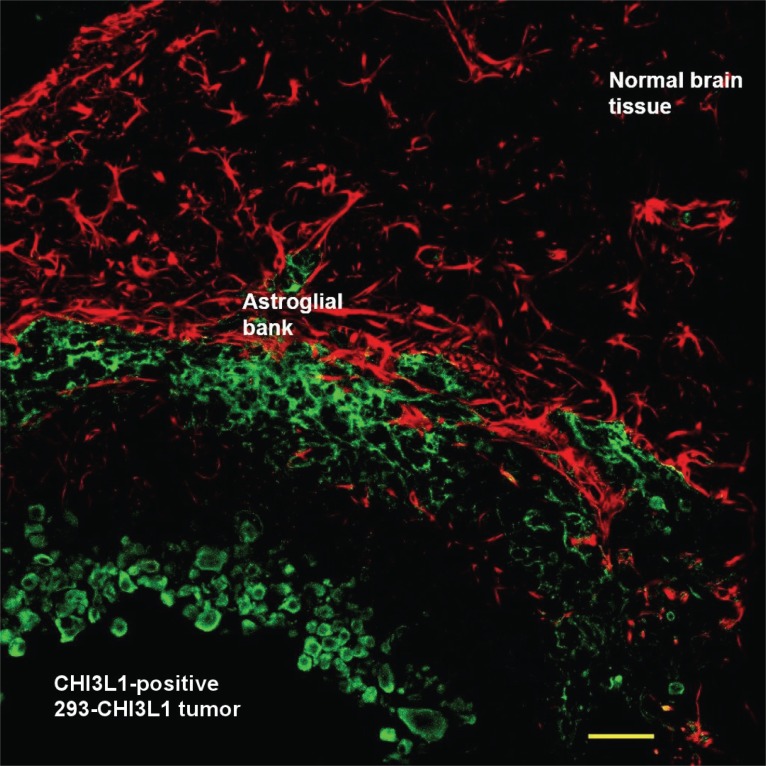
Immunofluorescence analysis of rat intracerebral tumor initiated by 293 cells stably expressing *CHI3L1*. Green fluorescence: anti-*CHI3L1* goat polyclonal antibodies and rabbit anti-goat Alexa Fluor 488. Red fluorescence: monoclonal antibodies to GFAP and rabbit anti-mouse Alexa Fluor 594. Yellow bar=50 microns.

In order to investigate CHI3L1 effect *in vivo*, 293 cells, which stably expressed *CHI3L1* or 293 cells transfected with empty vector, were stereotactically implanted in the caudoputamen of female Wistar rats (5 animals in each group). Tumor growth was monitored weekly *via* registration of neurological signs for up to 6 weeks. On the 21^st^ day after implantation severe neurological deficit appeared in 3 rats with implanted 293 cells, which stably expressed *CHI3L1.* These rats were killed and histological or immunohistochemical studies were performed. Huge intracerebral tumors were observed in each of these 3 rats (Fig. 6a). Histological assay revealed displacement of median cerebral structures and hydrocephalus in contralateral hemisphere. Tumors contained the dense superficial cell layer (Fig. 6b, green arrows) and prominent lobules (100-150 μm) with central newly ingrowing blood vessels (Fig. 6c, 6d). Immunostaining with anti-*CHI3L1* antibodies revealed *CHI3L1*-positive cells both in tumor tissue and in the border of tumor (an invasion zone). All tumors were surrounded by numerous GFAP-positive reactive astrocytes (Fig. 7).

The volume of gliomas estimated on serial tumor slices using the ellipsoid volume formula was 443.9, 575.7 and 523.5 mm3 for the first three rats (about 25% of the whole rat brain volume). Histological examination of remained two rats under investigation revealed tumors with extensive central necrosis as well, however, the tumor volume was about 300 mm3. No tumor growth was observed for at least 8 weeks in 5 rats injected with empty vector-transfected 293 cells as a negative control.

## DISCUSSION

Chitinase-like glycoprotein CHI3L1 has been investigated extensively with respect to its expression patterns and possible association with inflammation and cancer. A number of gene expression studies revealed *CHI3L1* as one of the most overexpressed genes in different tumors, basically on the last stages of tumor progression, particularly in glioblastoma (1-3 and present study) and is thought to regulate tumorigenesis by interrupting pathways which lead to apoptosis (20). *CHI3L1* expression was found in different cancer cells, tumor-associated macrophages and inflammatory cells and is involved in cell proliferation, differentiation, protection from apoptosis, angiogenesis, and invasiveness via remodeling of the extracellular matrix (4). The similarity between rat and human CHI3L1 is 80% (according to ClustalW2 results, http://www.ebi.ac.uk/Tools/msa/clustalw2/).

In the present study we showed that CHI3L1 had proliterative properties: 293 cells grow faster after addition of CHI3L1 to the culture medium. Moreover, 293 cells were oncogenically transformed by CHI3L1. These cells have increased capacity for anchorage-independent growth readily forming colonies in soft agar. In addition, *CHI3L1* overexpression in human 293 cells is highly efficient for inducing tumors in rat brain. Our data show that the overexpression of *CHI3L1* alone is sufficient to make 293 cells tumorigenic in Wistar rat brain, whereas no tumor was observed in animals injected with empty vector-transfected 293 cells. The immunohistochemical analysis of brain sections showed a significant increase in the GFAP expression in glioma compared to the normal hemisphere, same as it was observed in tumors, initiated by high grade glioma cultured cells (21).

Previously, primary rodent cells were efficiently converted into tumorigenic cells by the coexpression of cooperating oncogenes (22, 23). However, similar experiments with human cells have consistently failed to yield tumorigenic transformants, indicating a fundamental difference in the biology of human and rodent cells. The few reported successes in the creation of human tumor cells depended on the use of chemical or physical agents to achieve immortalization, the selection of rare, spontaneously arising immortalized cells, the use of an entire viral genome, or the ectopic expression of the telomerase catalytic subunit (hTERT) in combination with two oncogenes (the simian virus 40 large-T oncoprotein and an oncogenic allele of H-ras) (24). The conclusion of Ha *et al*. (2010) that human cervical cancer oncogene (*HCCR-1*) alone induced tumorigenic conversion of normal human cells (25) did not take into consideration that 293 cells themselves are immortalized already. 293 cells were generated in early 70s by transformation of cultures of normal human embryonic kidney cells with sheared adenovirus 5 DNA (26). Subsequent analysis has shown that the transformation was brought about by an insert consisting of ~4.5 kilobases from the left arm of the viral genome, which became incorporated into human chromosome 19 (27). Shaw *et al*. (28) provided evidence that 293 cells and several other human cell lines generated by adenovirus transformation of human embryonic kidney cells have many properties of immature neurons, suggesting that the adenovirus was taken up and transformed a neuronal lineage cell in the original kidney culture.

Here it is revealed *in vitro* and *in vivo* that ectopic expression of *CHI3L1* alone resulted in the direct tumorigenic conversion of immortalized 293 cells *in vitro* and *in vivo*, as it has been shown for *HCCR-1* (25), *PTTG1* (29), and *CnB* (30). Cells stably expressing *CHI3L1* had significantly increased ability for anchorage-independent growth in comparison with empty vector-transfected cells. Tumor formation by 293 stably expressing *CHI3L1* in rat brains strongly suggests that this gene is involved in oncogenesis and can be used as a target for anticancer drug development after understanding the mechanisms of CHI3L1 oncogenicity.

Immortality is an essential requirement for transformation that cooperates with other oncogenic changes to program the neoplastic state (31). Which mechanisms are responsible for malignant transformation of already immortalized 293 cells under the influence of CHI3L1 is a purpose of further investigations.
